# Tumor mutational signatures in sebaceous skin lesions from individuals with Lynch syndrome

**DOI:** 10.1002/mgg3.781

**Published:** 2019-06-04

**Authors:** Peter Georgeson, Michael D. Walsh, Mark Clendenning, Simin Daneshvar, Bernard J. Pope, Khalid Mahmood, Jihoon E. Joo, Harindra Jayasekara, Mark A. Jenkins, Ingrid M. Winship, Daniel D. Buchanan

**Affiliations:** ^1^ Colorectal Oncogenomics Group, Department of Clinical Pathology The University of Melbourne Parkville Vic. Australia; ^2^ Victorian Comprehensive Cancer Centre University of Melbourne Centre for Cancer Research Parkville Vic. Australia; ^3^ Sullivan Nicolaides Pathology Bowen Hills Qld Australia; ^4^ Dorevitch Pathology Frankston Hospital Frankston Vic. Australia; ^5^ Melbourne Bioinformatics The University of Melbourne Carlton Vic. Australia; ^6^ Cancer Epidemiology and Intelligence Division Cancer Council Victoria Melbourne Vic. Australia; ^7^ Centre for Alcohol Policy Research La Trobe University Melbourne Vic. Australia; ^8^ Centre for Epidemiology and Biostatistics, Melbourne School of Population and Global Health The University of Melbourne Carlton Vic. Australia; ^9^ Department of Medicine The University of Melbourne Parkville Vic. Australia; ^10^ Genomic Medicine and Family Cancer Clinic Royal Melbourne Hospital Parkville Vic. Australia

**Keywords:** Lynch syndrome, mismatch repair deficiency, mismatch repair immunohistochemistry, Muir‐Torre syndrome, sebaceoma, sebaceous adenoma, sebaceous carcinoma

## Abstract

**Background:**

Muir‐Torre syndrome is defined by the development of sebaceous skin lesions in individuals who carry a germline mismatch repair (MMR) gene mutation. Loss of expression of MMR proteins is frequently observed in sebaceous skin lesions, but MMR‐deficiency alone is not diagnostic for carrying a germline MMR gene mutation.

**Methods:**

Whole exome sequencing was performed on three MMR‐deficient sebaceous lesions from individuals with *MSH2* gene mutations (Lynch syndrome) and three MMR‐proficient sebaceous lesions from individuals without Lynch syndrome with the aim of characterizing the tumor mutational signatures, somatic mutation burden, and microsatellite instability status. Thirty predefined somatic mutational signatures were calculated for each lesion.

**Results:**

Signature 1 was ubiquitous across the six lesions tested. Signatures 6 and 15, associated with defective DNA MMR, were significantly more prevalent in the MMR‐deficient lesions from the *MSH2* carriers compared with the MMR‐proficient non‐Lynch sebaceous lesions (mean ± *SD*=41.0 ± 8.2% vs. 2.3 ± 4.0%, *p* = 0.0018). Tumor mutation burden was, on average, significantly higher in the MMR‐deficient lesions compared with the MMR‐proficient lesions (23.3 ± 11.4 vs. 1.8 ± 0.8 mutations/Mb, *p* = 0.03). All four sebaceous lesions observed in sun exposed areas of the body demonstrated signature 7 related to ultraviolet light exposure.

**Conclusion:**

Tumor mutational signatures 6 and 15 and somatic mutation burden were effective in differentiating Lynch‐related from non‐Lynch sebaceous lesions.

## 1 INTRODUCTION

Individuals who carry germline mutations in one of the DNA mismatch repair (MMR) genes (*MLH1*, *MSH2*, *MSH6,* or *PMS2*) have a high risk of developing cancer of the colon and rectum, and endometrium, among others, referred to as Lynch syndrome (Win et al., 2012). Muir‐Torre syndrome is a phenotypic variant of Lynch syndrome where individuals develop sebaceous skin lesions and internal (noncutaneous) malignancies (Lynch, Lynch, Pester, & Fusaro, 1981). One of the key mutational processes that is disrupted across cancers is the DNA MMR process, identified by tumor mutational signatures 6, 15, 20, and 26 (Wellcome Sanger Institute, 2019). Sebaceous neoplasms describe rare skin tumors involving the sebaceous glands that include sebaceous adenomas, sebaceous carcinomas, and sebaceomas (collectively referred to as sebaceous neoplasia in this study; reviewed in (Flux, 2017)). Lynch syndrome related tumors, including sebaceous skin lesions (Cesinaro et al., 2007), develop characteristic features namely widespread somatic DNA replication errors occurring within microsatellite repeats, referred to as microsatellite instability (MSI), and loss of expression of one or more of the MMR proteins determined by immunohistochemical (IHC) staining, collectively referred to as tumor MMR‐deficiency.

Recently, tumor mutational signatures have been identified that provide insights into the etiology of the tumorigenesis process through a genome‐wide assessment of somatic mutations and the sequence context in which they occur (Alexandrov et al., 2013). MMR‐deficiency in sebaceous neoplasia has been reported to be a sensitive screening tool for identifying MMR gene mutation carriers (Everett et al., 2014), however, the presence of MMR‐deficiency in a sebaceous lesion does not necessarily indicate the presence of a germline MMR gene mutation (Everett et al., 2014; Ponti et al., 2014; Roberts et al., 2014). The role of tumor mutational signatures in sebaceous neoplasia arising in the context of Lynch syndrome has not been well described. In this study, we report on whole exome sequencing (WES) derived tumor mutational signatures present in sebaceous skin lesions in people with and without Lynch syndrome.

## 2 METHODS

### 2.1 Study cohort

Individuals with sebaceous skin lesions were recruited to the Muir‐Torre study (Royal Melbourne Hospital and University of Melbourne Human Research Ethics Committee approval HREC/16/MH/8 and 1648355.1, respectively) from Sullivan Nicolaides Pathology and from the Australasian Colorectal Cancer Family Registry (University of Melbourne Human Research Ethics Committee approval 1339757 (Jenkins et al., 2018; Newcomb et al., 2007). Each participant's personal history of cancer and smoking were captured via a self‐reported questionnaire. Histopathological assessment (*SD*) determined sebaceous adenoma or carcinoma subtype. IHC testing for MMR protein expression identified MMR‐deficiency (loss of expression of one or more MMR proteins) or MMR‐proficiency (normal/retained expression of all four MMR proteins). Germline MMR gene testing was determined by gene specific Sanger sequencing and Multiplex Ligation Dependent Probe Amplification as previously described (Buchanan et al., 2017).

### 2.2 WES and loss of heterozygosity analysis

Formalin‐Fixed Paraffin Embedded (FFPE) tissue was macrodissected capturing areas of sebaceous differentiation. DNA extraction was performed using QIAamp DNA FFPE Tissue kit and standard protocols (Qiagen). Peripheral blood‐derived DNA was extracted using DNeasy blood and tissue kit (Qiagen) and sequenced as germline reference. Capture of the whole exome was performed using Agilent Clinical Research Exome V2 (Agilent) with sequencing performed on a NovaSeq 6000 comprising 150 bp paired‐end reads at targeted depths of 400 for FFPE tumor and 100 for buffy coat samples.

Adapter sequence was trimmed from the raw FASTQ files using trimmomatic 0.38, then aligned to GRCh37 using BWA 0.7.12. Somatic single‐nucleotide variants (SSNVs) were called with Mutect2, using recommended GATK practices, and Strelka 2.9.2 using Illumina's recommended workflow. Short insertions and deletions (indels) were called with Strelka 2.9.2 only. The intersection of SSNVs from these two callers was then limited to mutations with a minimum coverage depth of 40 reads and a minimum allele frequency of 10%. These filtered SSNVs were used to calculate mutational signatures using the Wellcome Trust Sanger Institute (WTSI) predefined set of 30 signatures (version 2) published on the COSMIC website. Each sample's set of mutational signatures was determined by computing the optimal linear combination of signatures that maximizes cosine similarity, as previously described by DeconstructSigs (Rosenthal, McGranahan, Herrero, Taylor, & Swanton, 2016). We assessed the impact of sequencing error due to DNA damage with Picard's CollectSequencingArtifactMetrics tool; all samples were considered to be of acceptable quality. MSI was determined using MSIsensor (Niu et al., 2014). Key driver SSNVs in the sebaceous lesions were considered to be those moderate and high impact variants, according to the Variant Effect Predictor, that occurred in somatic mutational “hotspots.” Somatic mutation hotspots were determined by recurrent mutation frequency in COSMIC mutation census. Details of the somatic pipeline are available at https://github.com/supernifty/somatic-pipeline.

Loss of heterozygosity (LOH) was determined by assessing regions of the genome containing heterozygous germline variants that appeared to be either homozygous reference or homozygous alternative in the somatic sample based on an allele frequency range of 0.3 to 0.7 in the germline variant and a difference of greater than 0.3 in the somatic variant. Details of the LOH algorithm are available at https://github.com/supernifty/loh_caller.

When referring to *MSH2* (OMIM: 609309) DNA changes and protein changes, the RefSeq transcripts are NM_000251.2 and NP_000242.1, respectively; the *HRAS* (OMIM: 190020) DNA change and protein change transcripts are NM_005343.2 and NP_005334.1 and the protein change transcripts for *EGFR* (OMIM: 131550), *MET* (OMIM: 164860), *RB1* (OMIM: 614041), and *POLE* (OMIM: 174762) are NP_005219.2, NP_001120972.1, NP_000312.2, and NP_006222.2, respectively.

Calculation of *p*‐values was performed using the two‐tailed unpaired student's *t* test.

## 3 RESULTS

**Figure 1:**
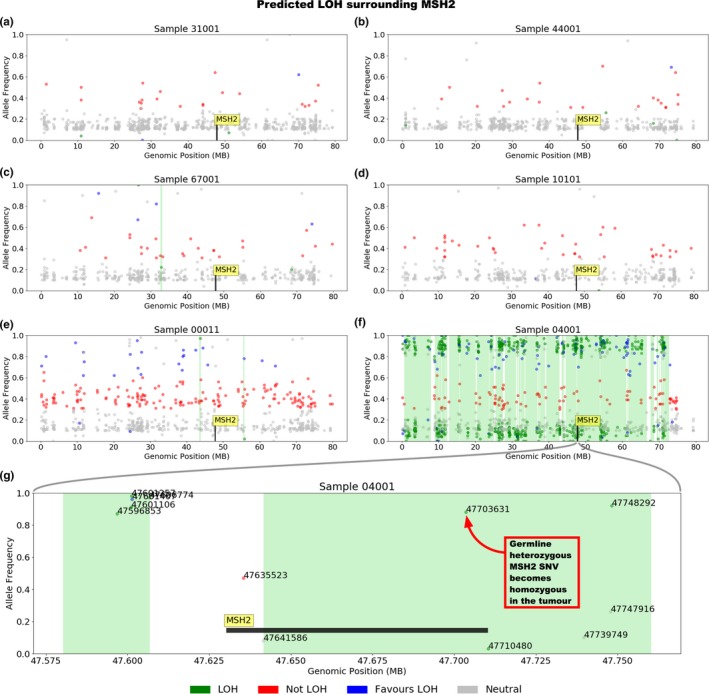
Plots (a‐f) depict the presence or the absence of loss of heterozygosity (LOH) in each tumor across the first 80 million bases of chromosome 2, which encompasses the *MSH2* gene. Samples 31001, 44001, 67001, 10101, 00011 show minimal LOH across this region, while 04001 demonstrates substantial evidence for LOH. Plot (g) shows *MSH2* and the immediately surrounding region in 04001, which demonstrates that a large proportion of the gene is affected by LOH, including the reported germline heterozygous mutation at c.2131

The characteristics of the individuals carrying germline MMR gene mutations in the *MSH2* gene (and MSH2/MSH6‐deficient sebaceous lesions) and individuals *without* germline MMR gene mutations (and MMR‐proficient sebaceous lesions), and their sebaceous lesions are described in Table 1. The average number of paired‐end reads for sebaceous lesions and blood‐derived DNA samples was 98,616,284 and 24,532,732, respectively, which translates to an average coverage of 298.2 and 81.1 across the target capture region. Interrogation of the WES confirmed the germline *MSH2* mutations in each of the three carriers (ID10101, ID00011, ID04001) and the absence of germline MMR gene mutations in the three noncarriers (ID31001, ID44001, ID67001). Sebaceous lesion tissue analysis revealed a “second hit” affecting the wild‐type allele for each of the three *MSH2* mutation carriers, confirming defects in both alleles and consistent with the loss of expression of MSH2 and MSH6 proteins in the tissue (Table 2). LOH across the *MSH2* gene region was observed as the second somatic hit in only one sample (Sample 04001; Figure 1).

**Table 1:** Clinico‐pathological characteristics of the six study participants

ID	Gender	Age at Diagnosis (years)^†^	MMR germline	Sebaceous Lesion	Site	MMR IHC status	Cancer history	Smoking history
*Individuals without Lynch syndrome*								
31001	M	64	None	Adenoma	R cheek	Normal expression	None	Former 20−38 years (infrequently)
44001	M	55	None	Adenoma	L temple	Normal expression	Skin cancer	Former 17−25 years
67001	M	70	None	Adenoma	L neck	Normal expression	Prostate (60 years); Melanoma (65 years)	Former (infrequently at school)
*Individuals with Lynch syndrome*								
10101	M	50	*MSH2* c.892C > T p.Gln298Ter	Adenoma	R nasal tip	MSH2/MSH6 loss	Colon (44 years); Ileocolon (54 years)	Former 10−48 years (Heavy)
00011	M	50	*MSH2* c.2131C > T p.Arg711Ter	Carcinoma	thigh	MSH2/MSH6 loss	Melanomas (50, 53, 60 years)	No
04001	F	64	*MSH2* c.2131C > T p.Arg711Ter	Carcinoma	back	MSH2/MSH6 loss	Renal pelvis (60 years); Colon (62 years); breast (70 years)	Current 30−64 years (moderate)

^†^ Age at diagnosis of lesion tested by whole exome sequencing; Gender: M = Male, F = Female; Site: L = left, R = Right side of body; MMR = mismatch repair; normal expression = normal and retained expression of all four MMR proteins tested (MMR‐proficiency), MSH2/MSH6 loss = loss of expression of MSH2 and MSH6 proteins with retained/normal expression of MLH1 and PMS2 proteins (MMR‐deficiency).

**Table 2:** Somatic mutation characteristics from the six sebaceous lesions tested by Whole Exome Sequencing

ID	Sebaceous lesion	Site	MMR germline	MMR IHC status	Somatic mutation characteristics				
					Somatic MMR mutation	Tumor mutational load	Tumor mutational burden (mutations/Mb)	Exonic microsatellites affected by indels	MSIsensor score (%)
31001	Adenoma	R cheek	None	Normal expression	None	184	2.7	13	0.34
44001	Adenoma	L temple	None	Normal expression	None	80	1.2	11	0.02
67001	Adenoma	L neck	None	Normal expression	None	100	1.5	15	0.24
10101	Adenoma	R nasal tip	*MSH2* c.892C > T p.Gln298Ter	MSH2/MSH6 loss	*MSH2* c.1216C > T p.Arg406Ter	943	14.0	139	2.54
00011	Carcinoma	Thigh	*MSH2* c.2131C > T p.Arg711Ter	MSH2/MSH6 loss	*MSH2* c.1861C > T p.Arg621Ter	1,341	19.9	555	21.11
04001	Carcinoma	Back	*MSH2* c.2131C > T p.Arg711Ter	MSH2/MSH6 loss	*MSH2* LOH c.942 onwards	2,421	36.0	674	23.53

Note

Tumor mutational load is the total number of SSNVs found by both Mutect2 and Strelka meeting minimum allele frequency and depth requirements and falling in the capture region. Tumor mutational burden is the number of SSNVs per megabase of capture region. Exonic microsatellites is the number of indels found in these regions by Strelka2. MSIsensor score is the percentage of microsatellites affected by indels according to MSISensor

Abbreviations: LOH, loss of heterozygosity; IHC, immunohistochemistry; MMR, mismatch repair.

The number of somatic mutations identified in the six sebaceous lesion exomes called by both variant callers and with sufficient allele frequency and read depth, and falling in the capture region, ranged from 80 to 2,421 which translates to tumor mutation burdens ranging from 1.2 to 36.0 mutations per megabase (mutations/Mb). The MMR‐deficient Lynch syndrome‐related lesions had, on average, significantly higher tumor mutation burden than the MMR‐proficient lesions (23.3 ± 11.4 vs. 1.8 ± 0.8 mutations/Mb per lesion, *p* = 0.03, Table 2). The two sebaceous carcinomas had the highest tumor mutation burdens (Table 2). Somatic indel mutations at exonic microsatellites were also enriched in the MMR‐deficient sebaceous lesions compared with the MMR‐proficient lesions (456 ± 280.9 vs. 13.0 ± 2.0; *p* = 0.052), consistent with a phenotype of replicative errors within short nucleotide repeat regions (microsatellite instability). The proportion of microsatellite sites affected by indels as measured by MSIsensor (Niu et al., 2014) was also higher in the MMR‐deficient samples compared with the MMR‐proficient samples (15.7 ± 11.5 vs. 0.2 ± 0.16; *p* = 0.08). Of note, all three of the MMR‐proficient sebaceous lesions acquired a predicted pathogenic somatic missense variant in *HRAS* (c.351G > T p.Lys117Asn, c.351G > T p.Lys117Asn and c.182A > G p.Gln61Arg in 31001, 44001, and 67001, respectively), with only MMR‐deficient sample 04001 acquiring a *HRAS* mutation (c.436G > A p.Ala146Thr). In addition, the MMR‐deficient samples acquired predicted pathogenic hotspot mutations; 10101 ‐ *EGFR* (p.Arg222Cys), 00011 ‐ *MET* (p.Ala320Val), and 04001 ‐ *RB1* (p.Arg556Ter) and *POLE* (p.Thr278Met), which were not observed in the MMR‐proficient samples. The MMR‐deficient samples acquired frameshift mutations in coding microsatellite regions that are frequently mutated in colorectal and/or endometrial cancer (Kloor & von Knebel Doeberitz, 2016) (Table 3).

**Table 3:** Genes with coding microsatellite regions that commonly have indel somatic mutations in colorectal and/or endometrial cancer reported in Kloor and von Knebel Doeberitz (2016)

Gene	MMR‐proficient Samples			MMR‐deficient Samples		
	31001	44001	67001	10101	00011	04001
*TGFBR2*	None	Frameshift	None	None	None	Frameshift
*TAF1B*	None	None	None	None	None	None
*AIM2*	None	None	None	Frameshift	None	None
*ASTE1*	None	None	None	None	Frameshift	Frameshift
*ACVR2*	None	None	None	None	Frameshift	None
*CASP5*	None	None	None	None	Frameshift	None
*NDUFC2*	None	None	None	Frameshift	None	None
*SLC2289*	None	None	None	None	Frameshift	Frameshift
*MSH3*	None	None	None	None	Frameshift	None
*SMAP1*	None	None	None	None	None	None
*OR7E24*	None	None	None	None	None	None
*KIAA2018*	None	None	None	None	None	Frameshift
*JAK1*	None	None	None	None	None	None
*C18ORF34*	None	None	None	None	None	Frameshift

The presence of a frameshift somatic mutation in the MMR‐proficient and MMR‐deficient sebaceous lesions is shown.

**Figure 2:**
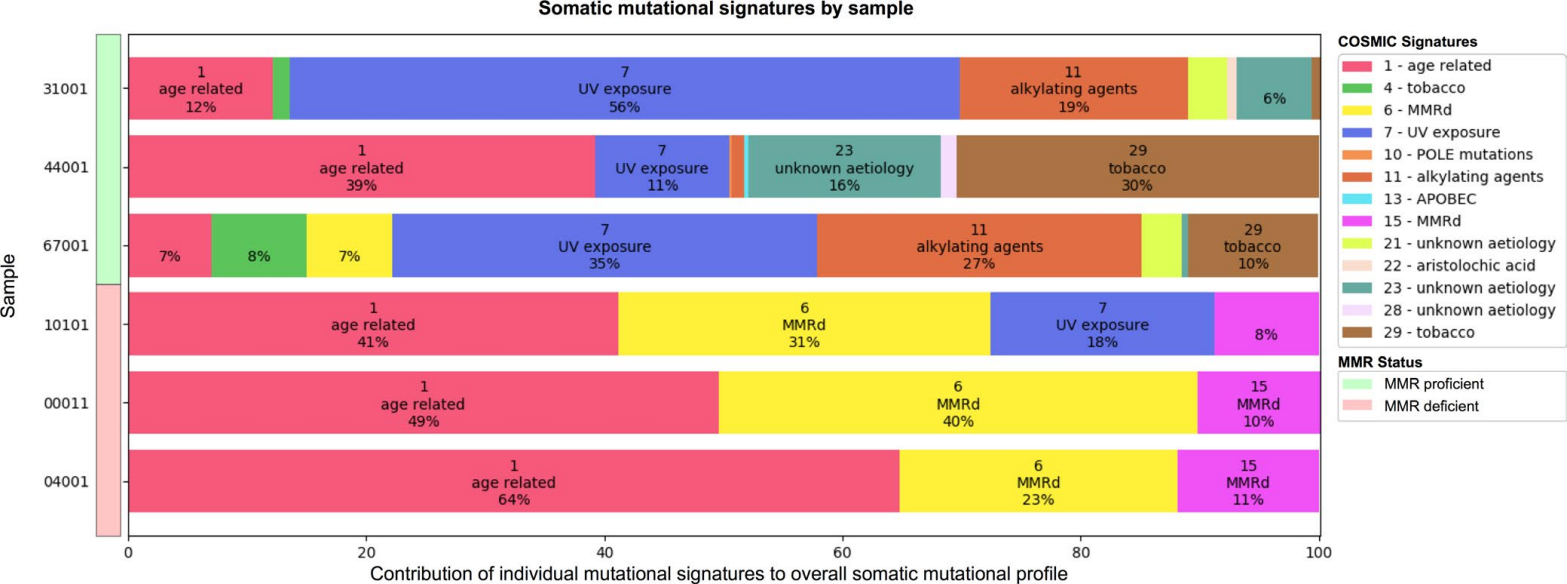
The mutational signature components for each of the six sebaceous lesions tested indicated by the signature number, suggested etiology and the percentage contribution to the overall mutational composition for each sample

The mutational signature composition for each of the six sebaceous lesions is shown in Figure 2. Two tumor mutational signatures related to defective DNA MMR, signatures 6 and 15, were evident in all MMR‐deficient samples. The MMR‐deficient Lynch syndrome related sebaceous lesions demonstrated a significantly increased proportion of mutational signature 6 compared with the MMR‐proficient sebaceous lesions, with a mean contribution within the overall signature composition of 31.3 ± 8.5% and 2.3 ± 4.0% per lesion, respectively (*p* = 0.006, Figure 2). Signature 15 was only observed in the MMR‐deficient lesions contributing on average 9.7 ± 1.5% per lesion to overall signature composition. The combined contribution of signatures 6 and 15 were 39%, 50%, and 34% for each of the three MMR‐deficient lesions with a mean ± *SD* of 41.0 ± 8.2% per lesion compared with combined 6 and 15 signatures in non‐Lynch affected MMR‐proficient samples of mean ± *SD* of 2.3 ± 4.0% per lesion (*p* = 0.0018).

Four of the sebaceous lesions developed on areas of the body typically exposed to greater amounts of sunlight (cheek, temple, neck, and nose), and concomitantly demonstrated the presence of signature 7 (Figure 2), whose etiology is related to ultraviolet‐associated DNA damage. The other two sebaceous lesions that occurred on the thigh and back did not demonstrate signature 7. The sebaceous adenoma from person 44001 demonstrated a tobacco‐related signature, signature 29, and comprised ~30% of the overall signature composition. This individual reported smoking for a period of 8 years, some 30 years prior to the removal of the sebaceous adenoma at age 55 years. The MMR‐proficient sebaceous adenomas from participants 31001 and 67001 demonstrated less dominant tobacco‐related signatures, signatures 4 and 29. Both participants were former smokers reporting infrequent smoking behavior. In contrast, none of the MMR‐deficient sebaceous lesions showed a tobacco‐related signature despite two of the three *MSH2* carriers reporting moderate to heavy smoking for over 30 years.

## 4 DISCUSSION

Tumor mutational signatures are derived from the type of somatic base substitution and the sequence context in which they occur, providing novel insights into the biological processes operative in the development and progression of the tumor (Alexandrov et al., 2013). In this study, the sebaceous lesions from those individuals with a *MSH2* germline mutation and an identified second somatic hit of the wild‐type allele (Lynch syndrome) demonstrated a significantly higher tumor mutational burden and number of somatic indels at exonic microsatellites, compared with the MMR‐proficient non‐Lynch sebaceous lesions. These characteristics are associated with MMR‐deficiency in other Lynch syndrome‐related cancer types (Cortes‐Ciriano, Lee, Park, Kim, & Park, 2017). In addition, we observed a higher enrichment of tumor mutational signatures 6 and 15 in these samples, compared with the MMR‐proficient non‐Lynch sebaceous lesions. Signatures 6 and 15 have been previously associated with defective DNA MMR in other cancer types (Alexandrov et al., 2013; Ma, Setton, Lee, Riaz, & Powell, 2018). Signatures 6 and 15 together contributed on average 41% of the overall signatures in the three MMR‐deficient lesions, and is consistent with a previous study which demonstrated a significant contribution of signatures 6 and 15 to the overall signature composition in one individual with Lynch syndrome who developed a MSI‐H (MMR‐deficient) sebaceous carcinoma (North et al., 2018). These findings highlight the dominant association that loss of MMR function has with the development of sebaceous neoplasia in people with Lynch syndrome.

Of interest, signatures 20 and 26, have been associated with defective MMR (Helleday, Eshtad, & Nik‐Zainal, 2014; Temko, Tomlinson, Severini, Schuster‐Bockler, & Graham, 2018; Wellcome Sanger Institute, 2019), but were not observed in the Lynch‐related sebaceous skin lesions in this study. Only three MMR‐deficient sebaceous lesions were tested in this study and so they may not represent the complete heterogeneity of mutation signatures that are observed in Lynch‐related sebaceous neoplasia. In support of our findings, signatures 20 and 26 were not observed in the nine MSI‐H (MMR‐deficient) sebaceous carcinomas in the study by North and colleagues (North et al., 2018) suggesting these two defective MMR signatures are not significant indicators of sebaceous neoplasia development. In addition, signatures 20 and 26 are generally less commonly observed. Across the TCGA cohort (Tomczak, Czerwinska, & Wiznerowicz, 2015), signatures 6, 15, 20, and 26 are observed (at a level greater than 10%) in 23%, 15%, 7%, and 3% of samples, respectively.

Signature 1 was present in all six sebaceous lesions, observed as the dominant signature in 4/6 sebaceous lesions tested. Signature 1 is a consequence of deamination of 5‐methylcytosine, which is not only associated with age but also formalin fixation (Do & Dobrovic, 2015). We assessed the impact of sequencing error due to FFPE‐derived DNA damage (see Methods), however, all samples were considered to be of acceptable quality. Signature 1 has been commonly found in all cancer types including in Lynch syndrome related sebaceous neoplasia (North et al., 2018).

All four of the sebaceous adenomas tested in this study were on the head and neck region and demonstrated an ultraviolet damage mutational signature (Signature 7). This signature was absent in the two sebaceous carcinomas that occurred on the thigh and back, areas less likely to be sun exposed. In comparison, (North et al., 2018) observed signature 7 in 10/14 of the sebaceous carcinomas tested on the head and neck region, including in one of the MSI‐H sebaceous carcinomas, supporting our findings and the significance of an ultraviolet DNA damage mutational process in sebaceous lesion development in sun exposed areas. One MMR‐deficient sebaceous adenoma in our study (sample 10101) demonstrated both MMR‐deficiency (signatures 6 and 15) and ultraviolet damage (signature 7) signatures, the contribution of signatures 6 and 15 to the overall signature composition was greater than that of signature 7 (39% vs. 18%). Similarly, North et al. (2018) observed a single MMR‐deficient sebaceous carcinoma from a non‐Lynch person that exhibited both a MMR‐deficiency (signatures 6 and 15) and an ultraviolet damage (signature 7) signature where the contribution of the defective MMR signatures was also greater than the ultraviolet damage signature. Loss of MMR function appears to be a more dominant driver of tumorigenesis in sebaceous neoplasia even in sun exposed areas of the body although further support for this is needed.

The MMR‐proficient ultraviolet‐damaged related sebaceous carcinomas from North et al. (2018) showed the highest number of SSNVs even compared with the MMR‐deficient sebaceous carcinomas. In our study, the three MMR‐proficient tumors exhibited substantially lower somatic mutational burden than the MMR‐deficient tumors, both for exonic SSNVs (average 121 and 1,568, respectively) and exonic microsatellite indels (average 13 and 456, respectively).

We also investigated the relationship between smoking status and the presence of mutational signatures 4 and 29. These two signatures have been associated with DNA damage due to tobacco mutagens (Alexandrov et al., 2016; Wellcome Sanger Institute, 2019). Signature 29 was observed in all three of the MMR‐proficient sebaceous adenomas, where all three people reported a previous history of smoking. Interestingly, signature 29 was most prominent in MMR‐proficient sebaceous adenoma from 44001 who reportedly stopped smoking 30 years before the diagnosis of the adenoma. None of the MMR‐deficient sebaceous lesions samples exhibited signature 29 despite one reporting he was a former heavy smoker (person 10101) while person 04001 was still smoking at the time of sebaceous carcinoma diagnosis. The absence of signature 29 in these individuals suggests that the loss of MMR function could be a more significant contributor to the development and progression of their sebaceous lesions than effects from tobacco mutagens. It is also possible that the MMR‐deficient sebaceous lesions have originated before the exposure to tobacco mutagens. However, for each of the six people in this study we have no information on exposure to passive smoking or occupational exposure to tobacco mutagens which may influence the presence of signature 29.

## 5 CONCLUSIONS

MMR‐deficient sebaceous lesions from *MSH2* gene mutation carriers demonstrated a significantly higher tumor mutation burden, increased numbers of indels in exonic microsatellite repeats and the highest proportion of tumor mutational signatures 6 and 15 compared with MMR‐proficient non‐Lynch sebaceous lesions, features which are consistent with other cancer types demonstrating MMR‐deficiency. Ultraviolet‐related signatures were important contributors to the mutational process in sebaceous lesions on sun exposed areas of the body including in the presence of MMR‐deficiency. Tobacco‐related signature 29 was only observed in MMR‐proficient sebaceous samples in our study and requires further investigation to elucidate its contribution to the oncogenic processes in sebaceous neoplasia development. Here, we demonstrated the utility of assessing tumor mutational signatures and tumor mutational burden for differentiating MMR‐deficient Lynch‐related from non‐Lynch MMR‐proficient sebaceous lesions which may facilitate improved identification of high‐risk MMR gene mutation carriers.

## CONFLICT OF INTEREST

The authors have no conflict of interest.

## DISCLOSURE

Dr Buchanan served as a consultant on the Tumor Agnostic (dMMR) Advisory Board of Merck Sharp and Dohme in 2017 and 2018 for Pembrolizumab.
